# Emerging scientists in analytical sciences: Kim Greis

**DOI:** 10.1002/ansa.202200036

**Published:** 2022-10-21

**Authors:** Kim Greis

**Affiliations:** ^1^ Department of Molecular Physics Fritz Haber Institute of the Max Planck Society Berlin Germany; ^2^ Institute of Chemistry and Biochemistry Freie Universität Berlin Berlin Germany

**Abbreviations**
IMS: ion mobility spectrometry
RNA: ribonucleic acid
m/z: mass‐to‐charge
MS: mass spectrometry
SLIM: structures for lossless ion manipulations

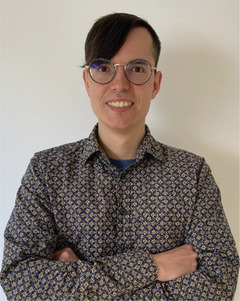


**Introduction**

Kim Greis is a third‐year PhD candidate in the lab of Prof. Kevin Pagel at Freie Universität Berlin and Fritz Haber Institute of the Max Planck Society. He joined Humboldt‐Universität zu Berlin in 2014 for his bachelor's studies in chemistry and stayed there for his master's degree. During the latter, he went as an exchange student to the University of Melbourne for a research stay. In 2019, he switched to Freie Universität Berlin to start a PhD project in the group of Prof. Kevin Pagel to study reactive intermediates from bioorganic reactions using mass spectrometry‐based methods and density‐functional theory calculations. Despite his young career he has already published 21 papers (and counting) and collected numerous prestigious awards, such as a Fulbright Grant, which allowed him to do research at Yale University during his PhD.

1

**How did you get involved in the field of analytical sciences?**

I did my bachelor's and master's studies at the Humboldt‐Universität zu Berlin. In contrast to other chemistry departments, there is a big analytical division at Humboldt‐Universität zu Berlin. Hence, a large selection of mandatory analytical chemistry courses was available. For my bachelor thesis, I joined the lab of Prof. Klaus Rademann and developed a cellulose‐based sensor to detect low concentrations of toxic metal ions in aqueous solutions.^1^ During my master's studies, I switched fields during an exchange internship at the University of Melbourne, where I joined the lab of Prof. Richard O'Hair. Here, I got hands‐on experience with mass spectrometers for the first time. We used a modified ion trap mass spectrometer that allows for ion‐molecule reactions to study phenanthroline‐ligated transition state metal complexes. With this setup, we got information on the reactivity of these species and in some cases, we were able to reveal catalytic cycles.^2^, ^3^ Furthermore, I learned in Australia about using computational methods, such as density‐functional theory, to support my data from mass spectrometry. Subsequently, I joined the lab of Kevin Pagel at Freie Universität Berlin and the Fritz Haber Institute of the Max Planck Society for my master's thesis and stayed there for my PhD.

2

**What is the topic of your PhD studies?**

In my PhD studies, I combine computational methods and cryogenic vibrational spectroscopy in helium nanodroplets to investigate the structure of reactive intermediates. Cryogenic vibrational spectroscopy of ions is a mass spectrometry‐based technique that I will introduce in a moment. The method yields highly resolved infrared spectra that, in combination with computed frequencies, are very suitable to determine the structure of ions. We used the technique mainly to study glycosyl cations, the reactive intermediate of glycosynthesis.^4^, ^5^, ^6^ In a second step, we correlate the structure with the stereoselectivity that can be observed in condensed‐phase glycosylation reactions. We found that there is a correlation between the gas‐phase structure and the condensed‐phase stereoselectivity. Recently, I also investigated intermediates from RNA autohydrolysis and smaller carbocations with this method.^7^

3

**Which technologies are you using in your laboratory?**

In our lab, we use different techniques such as high‐performance liquid chromatography, ion mobility–mass spectrometry, cryogenic infrared spectroscopy (in helium droplets and with messenger‐tags) and computational methods. For my project, I am mainly focusing on cryogenic infrared spectroscopy in helium droplets and computational methods. Therefore, I use a custom‐built setup that was developed at the Fritz Haber Institute by the group of Prof. Gert von Helden. Here, the probed ions are generated by nano‐electrospray ionization and can be fragmented using in‐source fragmentation. A quadrupole is used for *m*/*z* selection, after which the selected ions are stored in a hexapole ion trap. A beam of superfluid helium nanodroplets (0.4 K) is guided through the ion trap and picking up the ions of interest, cooling them to 0.4 K and guiding them out of the trap, where they overlap with an infrared laser generated by the in‐house Fritz Haber Institute free‐electron laser. Absorption of multiple resonant photons leads to the release of the ions from the droplets, an event that can be monitored with a time‐of‐flight detector. Plotting the ion signal as a function of the photon wavenumber yields an infrared spectrum.

4

**What was your biggest achievement during your PhD time?**

One year into my PhD studies, I published a paper on the structure of the Ferrier cation.^5^ This ion is the intermediate of the Ferrier rearrangement reaction. In many textbooks and past publications, it is claimed that the charge in this ion is stabilized by delocalization within the pyranose ring. A smaller fraction of publications hypothesized the existence of a structure in which the charge is stabilized by neighbouring group participation of an acetyl group. Based on our experiments, we found out that only the latter exists. This example nicely shows that the structures of species that are postulated are not necessarily corresponding to our first intuition. I hope that these results will convince editors to revise the textbooks.

5

**As part of your PhD programme you went to the united states. Could you give us more details about your research stay?**

I went to the United States as a Fulbright scholar. Here, I was a visiting assistant in research at Yale University in the group of Prof. Mark Johnson for 6 months. At Yale, I did experiments using their messenger‐tagging cryogenic infrared spectroscopy setup and supported the research group using computational methods. During my stay, I got insight into how this technique can be used not only to study the structure of ions but also to gain insight into the chemical reactivity of isolated, sometimes microsolvated complexes in the gas phase. Furthermore, Mark Johnson taught me to look differently at my results, so that I can extract as much knowledge from them as possible, without relying too much on computations. Although this research stay only happened earlier this year, two papers with results are already published.^8^, ^9^ Besides the excellent academic environment, I also enjoyed being a member of the Yale Tango Club, which was a strong social network for me during my time in the United States.

6

**What advice would you give to new PhD candidates?**

First, I would recommend aspiring PhD candidates to work on a project that really sparks their interest. If it does not excite you, it will be very hard working for three or more years in the field and write a whole thesis on it. Once you started your PhD work, be open to new ideas and collaborations. It is always beneficial when you try to think about how your methods could benefit other projects or vice versa. Attend conferences to absorb knowledge and do not be afraid to network with potential supervisors or collaborators.

7

**What do you plan to do after your PhD studies?**

After my PhD studies, I plan to do a postdoctoral stay in a lab, where I can combine mass spectrometry‐based techniques with computational methods. It is important to me that I will have the opportunity to taste a different flavour of mass spectrometry than what I am used to, without straying too far off my current path. I plan that the acquired knowledge in combination with my current skills prepares me well for working as an independent group leader afterwards.

8

**Did you feel well‐prepared for your PhD studies and would you suggest changes for the academic curriculum?**

Personally, I felt well prepared. This is mainly due to the very intense bachelor's course at Humboldt‐Universität zu Berlin. In my master's studies, I would have wished for more flexibility so that I could have deepened my knowledge in relevant fields. I was lucky that I did the research stay in Prof. Richard O'Hair's group, which prepared me well for everything that followed afterwards. So generally, I would suggest that academic curricula should allow for more flexibility and encourage students to do more internships in research groups and/or companies.

9

**Which current trends in analytical instrumentation for mass spectrometry are you interested in?**

Recently, there have been many interesting developments in mass spectrometry. I am personally very interested in the Waters Cylic IMS and the MOBILion SLIM.^10^, ^11^ Especially the recent advances in the SLIM technology by Rizzo et al. would be immensely useful if commercialized.^12^, ^13^ What I am currently interested the most in is the Omnitrap developed by Fasmatech.^14^ This platform essentially allows you to use all fragmentation techniques in an MS*^n^* manner to characterize your analyte ions and is hence very powerful. I hope that I will someday be able to work on a mass spectrometer that has the Omnitrap!

10

**Where do you see yourself in 10 years?**

In 10 years, I see myself researching fundamental ion chemistry. I want to use mass spectrometry‐based methods to provide an in‐depth understanding of the structure and reactivity of ionic species. Ultimately, I want to use that knowledge to optimize existing and design new chemical reactions that will aid to transform the chemical industry into a sustainable circular economy.


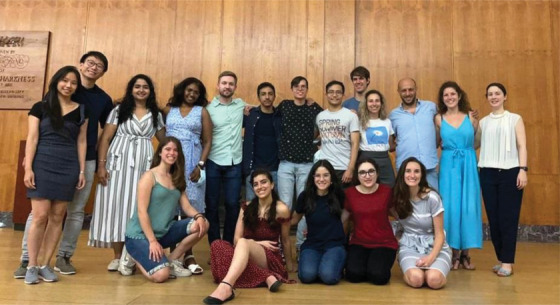


11

**Can you say something about your hobbies outside the laboratory?**

Whenever I get some time off work, I like meeting friends and hiking in nature. Some of my friends would probably say that I have a strong interest in eating cookies and watching videos of cats. This fall, I found interest in collecting edible mushrooms from the forest. Occasionally, I watch movies and play video games. Earlier this year, I started learning Chinese and dancing Argentinian Tango. I picked up the latter while I was in the United States, where we met at least twice a week do dance and every now and then went to social dance events, so‐called Milongas!

## CONFLICT OF INTEREST

The author declares no conflict of interest.
